# Important Considerations for Examining Endothelial Dysfunction in Rheumatoid Arthritis

**DOI:** 10.31138/mjr.28.3.112

**Published:** 2017-09-29

**Authors:** Aamer Sandoo

**Affiliations:** 1 School of Sport, Health and Exercise Sciences, Bangor University, Bangor, Wales, United Kingdom,; 2 Dudley Group of Hospitals NHS Trust, Russells Hall Hospital, Dudley, United Kingdom

**Keywords:** Endothelium, atherosclerosis, microvascular, cardiovascular disease, rheumatoid arthritis

Rheumatoid arthritis (RA) is the most common inflammatory autoimmune disease with patients presenting with an increased risk for cardiovascular disease (CVD).^1^ It has been hypothesised that interactions between RA disease-related inflammation and classical cardiovascular disease risk factors may accelerate atherosclerosis.^2^ Examination of cardiovascular risk is typically performed using non-invasive assessments of vascular function and structure in the peripheral microvessels and the large vessels (**Figure 1**).^3^ These assessments correlate well with invasive assessments in the coronary circulation,^3^ and can predict worse cardiovascular outcomes in the general population.^4^ In contrast, there are limited studies examining the predictive value of vascular assessments on adverse cardiovascular outcomes in RA, and several other issues need to be considered when incorporating such assessments in future research. The present editorial attempts to outline these issues and explain how they might be addressed in future research studies.

**Figure 1:**
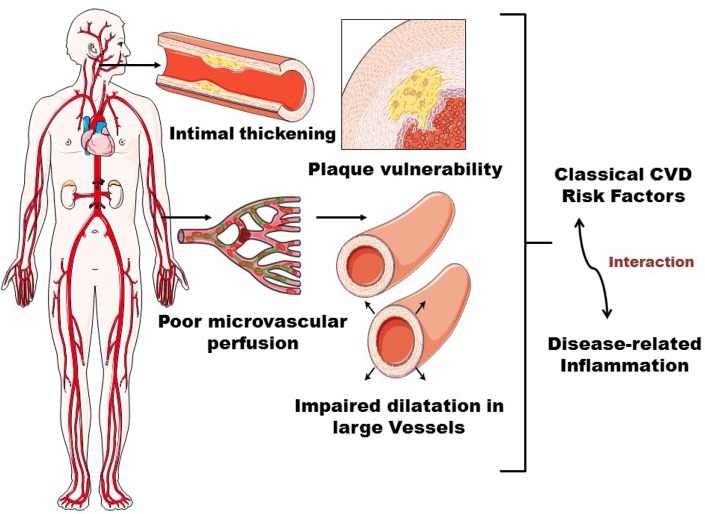
Non-invasive assessments can be utilised in the carotid artery to provide information about intimal thickening as well as plaque stability. Assessments in the microcirculation and large vessels indicate the functional response of the endothelium to physiological and pharmacological stimulation. The majority of studies in rheumatoid arthritis report that classical CVD risk factors and inflammation interact with each other to adversely impact on the vasculature.

## ENDOTHELIAL DYSFUNCTION IN RA: A FOCUS ON INFLAMMATION AND CLASSICAL CVD RISK FACTORS

It has long been hypothesised that RA disease-related inflammation impacts on the vasculature in RA.^5^ However, evidence supporting this hypothesis was largely assumed from studies showing an improvement in vascular function following anti-inflammatory treatment,^6^ as well as some (mainly cross-sectional) studies reporting associations between systemic inflammatory markers and the vasculature.^7^ In our systematic review conducted in 2011, the majority of cross-sectional and longitudinal studies did not show consistent associations between disease-related inflammation and the vasculature.^8^ Furthermore, of the 33 studies that longitudinally examined changes in the vasculature following anti-inflammatory treatment, only one revealed that improvement in arterial stiffness was associated with a reduction in systemic inflammation.^9^ In a series of studies from our own group, RA disease related inflammation did not associate with vascular health in a large sample (n = 99) of well-characterised RA patients examined cross-sectionally, and in 23 patients who received anti-tumour necrosis factor-alpha and were longitudinally followed-up for 1 year.^10^ Subsequent studies revealed that in RA patients prospectively followed-up for six years, the baseline factors most likely to predict adverse vascular function and morphology included classical CVD risk factors such as hypertension, dyslipidemia and insulin resistance, but not RA disease-related inflammation.^11^

Interestingly, erythrocyte sedimentation rate (ESR) and C-reactive protein (CRP) measured cumulatively over the 6 year period did not associate with parameters of vascular function, but a weak association was evident between cumulative CRP and carotid atherosclerosis.^11^ In a more recent study examining predictors of carotid atherosclerosis in a large sample of RA patients, cumulative ESR and the number of classical CVD risk factors both interacted with each other to associate with the presence of carotid plaque (an advanced, yet subclinical marker of atherosclerosis).^12^ In another study, ESR only associated with carotid intima-media thickness in the presence of classical CVD risk factors.^13^ Finally, large-vessel endothelial function assessed using flow-mediated dilatation (FMD) was not different between RA and type II diabetics (the archetypal condition for the development of CVD), despite RA patients having greater CRP concentrations.^14^ In sum, the use of systemic inflammatory markers (which primarily reflect joint inflammation) to predict adverse vascular outcomes do not adequately reflect changes in the vascular wall, and consideration should be given to the interplay between classical CVD risk factors and vascular wall inflammation in potentiating vascular damage.

In RA, the prevalence of classical CVD risk factors such as hypertension and dyslipidemia are increased, and their control is often worse when compared to the general population.^15^ Unfortunately, with the exception of the research mentioned above, very few studies have directly examined the impact of classical CVD risk factors on vascular health in RA. The strongest evidence comes from the prospective randomised control trial by Metsios et al.^16^ who reported that in previously sedentary RA patients, six months of aerobic and resistance exercise significantly reduced the presence of several CVD risk factors, improved cardiorespiratory fitness (a strong indicator of overall cardiovascular health) and improved endothelial function in the microvessels and large vessels.^17^ These findings highlight the importance of adopting non-pharmacological interventions which exert direct beneficial effects on the vasculature leading to reduced CVD risk. Further, they highlight that targeted reduction of CVD risk should include aggressive management of classical CVD risk factors.

## THE CONCEPT OF ACCELERATED ATHEROSCLEROSIS AND RISK OF CARDIAC EVENTS

Rheumatoid arthritis patients are postulated to have accelerated atherosclerosis, yet at present; there is very little strong evidence for this. Nagata Sakurai et al.^18^ noted greater carotid intima-media thickness (cIMT) in 62 female RA patients relative to age-matched female healthy controls, and reported an association between baseline CRP concentration and increase in cIMT following a 1.5–3-year follow-up. However, the relationship between change in inflammation and cIMT could not be characterised due to the absence of CRP measurement at follow-up visits. The ability of non-invasive assessments to predict future CVD risk in RA is also presently unclear. To date, only one study with a small sample size (N=47) revealed that over a 5-year follow-up period, the 8 patients who experienced a cardiac event had greater cIMT at baseline than those patients who did not have a cardiac event.^19^ It is noteworthy to mention that patients who experienced a cardiac event tended to be older, the proportion of cardiac events overall was small, and cIMT was not assessed at the end of the 5-year follow-up. Thus, while non-invasive assessments of vascular health can predict cardiac events in the general population,^4^ prospective studies specifically in RA are warranted. These studies should prospectively examine occurrence of cardiac events over a long follow-up period, examine vascular health in different vascular beds and control for multiple determining factors.

RA patients also present with increased plaque formation which are typically inflamed and unstable in nature.^20^ An autopsy study conducted by Aubrey and colleagues^21^ revealed that RA patients had greater unstable and inflamed plaques than age- and gender-matched controls, despite having less severe stenosis than controls. In another study, patients with various types of inflammatory arthritis including RA tended to have greater inflammatory insult in the aortic wall when compared to age- and gender-matched controls.^22^ Indeed, coronary artery disease is often silent in RA, with a higher proportion of unrecognised myocardial infarction and sudden cardiac death.^23^ Thus, case fatality is likely to be linked to the sudden rupture of unstable plaques rather than *accelerated* atherosclerotic narrowing of the vessel. Importantly, the predictors for the development of new carotid plaques in RA appear to be similar to predictors of endothelial function and include the presence of classical CVD risk factors rather than systemic disease-related inflammation.^24^

## THE IMPORTANCE OF MICROVASCULAR ENDOTHELIAL FUNCTION

The microvessels differ quite markedly to the large vessels in anatomical structure and physiological function and often display heterogeneous blood flow responses when stimulated.^25^ Indeed, we have previously reported that endothelium-dependent and endothelium-independent responses in the microvessels do not associate with large vessel responses in RA.^26^ In the coronary circulation, RA patients often exhibit poor cardiac perfusion despite clear epicardial arteries, which is indicative of poor coronary microvascular function.^27^ In addition, surrogate measures of myocardial ischemia such as the subendocardial viability ratio are adversely affected by RA disease-related inflammation and classical CVD risk factors.^28,29^ It is worth noting that the vascular profile and CVD risk in RA is similar to that in type II diabetes,^14^ a condition in which microvascular disease often contributes to large vessel atherosclerosis.^30^ Unfortunately, only a small number of studies have actually examined microvascular endothelial function in RA, and many utilise different vascular techniques resulting in heterogeneous findings.^8^ The microvessels possess a much greater surface area than the large vessels and are likely to be more susceptible to the injurious effects of classical CVD risk factors and inflammation.^31^ Thus, there is a need for greater studies examining specific RA-related determinants of microvascular endothelial function. In particular, studies should attempt to characterise whether microvascular endothelial function is a strong predictor of future cardiac events in RA.

## FUTURE DIRECTIONS

The available literature clearly shows that RA patients have worse vascular health relative to healthy controls. However, the precise determinants of adverse vascular outcomes are not clear, with the majority of studies not supporting a relationship between RA-related systemic inflammation and the vasculature. In fact, it is clear that classical CVD risk factors exert the strongest influence on the vasculature directly and via subtle interactions with RA disease-related inflammation. Importantly, while non-invasive assessments of endothelial function can predict cardiac events in the general population, it is presently unclear which assessments provide an index of future CVD risk in RA, if at all. This important question needs to be answered by prospectively examining several assessments of the vasculature in large well-characterised RA cohorts (possibly from multicentre studies) who are followed up over a long period of time. Any such study should include assessments of microvascular endothelial function and structure, owing to the fact that these small vessels make up a large surface area and are often the first to be damaged by CVD risk factors. Indeed, it has been hypothesised that the microvessels are the proponents of large vessel disease in populations at high risk of CVD.^31^
